# Agricultural propiconazole residues promote triazole cross-resistance in *Cryptococcus neoformans* through *ERG11* and efflux pump overexpression

**DOI:** 10.1128/aac.00765-25

**Published:** 2025-07-31

**Authors:** Hantao Yu, Wenwen Peng, Hang Li, Ying Zhang, Xiaoxiang Fu, Xia Gong, Hongyi Wei, Qinghong Zhou, Yingjin Huang, Duantao Cao

**Affiliations:** 1 The Laboratory for Phytochemistry and Botanical Pesticides, College of Agriculture, Jiangxi Agricultural University 91595, Nanchang, China; 2 Jiangxi Province Key Laboratory of Vegetable Cultivation and Utilization, Jiangxi Agricultural University 91595, Nanchang, China; 3 College of Chemistry and Materials, Jiangxi Agricultural University 91595, Nanchang, China; University Children's Hospital Münster, Münster, Germany

**Keywords:** *Cryptococcus neoformans*, propiconazole, resistance, triazole, soil

## Abstract

Fluconazole remains a cornerstone for consolidation therapy in cryptococcal meningitis. However, resistance poses a significant risk of treatment failure in sub-Saharan Africa, where fluconazole monotherapy is common. Beyond clinical use, triazole fungicides are suspected drivers of resistance pathways. Nevertheless, there is limited evidence linking triazole fungicide use to the emergence of resistant *C. neoformans* (RCN) in soil. Here, the possibility of evolving triazole resistance in *C. neoformans* by exposing it to the triazole fungicide propiconazole was explored. The findings suggest that propiconazole can induce the resistance of *C. neoformans* to triazole drugs in liquid media and soil by upregulating the expression levels of target gene *ERG11* and efflux pump genes *AFR1*, *AFR2*, and *AFR3*. Interestingly, there were 2, 5, 4, and 3 RCN strains isolated from propiconazole-treated soils at 1, 2, 5, and 10 mg/kg, respectively, indicating that the emergence of RCN isolates depends on the residual concentrations of propiconazole. These findings suggest that the field application of propiconazole, even at the recommended dosage, can promote the development of triazole resistance in *C. neoformans*, thereby affecting the efficacy of triazole drugs and hindering the prevention and management of cryptococcosis.

## INTRODUCTION

*Cryptococcus neoformans* is the main pathogen responsible for severe cryptococcosis that encompasses conditions, such as pneumonia and cryptococcal meningitis, and primarily infects immunodeficient populations, with approximately 1 million new cases per year and a mortality rate of 30%–70% (1–3). The recommended cryptococcosis regimen relies on amphotericin B plus flucytosine despite their substantial toxicity and limited availability, followed by fluconazole (FLU) consolidation therapy. Other triazoles like itraconazole (ITR), voriconazole (VOR), and posaconazole (POS) may be considered in cases of FLU intolerance, resistance, or as salvage therapy for refractory infections (4–6). However, in many resource-limited settings, particularly across sub-Saharan Africa, FLU serves as the sole available and affordable antifungal agent, necessitating its use for the entire treatment course of cryptococcosis. This heavy reliance on FLU monotherapy, driven by limited access to optimal therapeutics like amphotericin B and flucytosine in high-burden regions, significantly complicates disease management and poses a potential risk of treatment failure due to the emergence of resistance (7–9).

The main mechanisms that cause resistance of *C. neoformans* to triazole medicines include the following: (i) amino acid substitutions in the coding region of the target gene *ERG11* affect the binding of triazoles to the 14α-demethylase enzyme, for example, the Y145F mutation leads to strains showing resistance to FLU and VOR (10), the G484S mutation leads to FLU resistance (11, 12); (ii) heteroresistance, a crucial adaptive mechanism in *Cryptococcus* under azole pressure (13, 14); (iii) elevated intracellular levels of target enzymes due to *ERG11* overexpression (15, 16); (iv) overexpression of efflux pump genes in ABC (ATP binding cassette) and major facilitator superfamily (MFS) transporters, such as *AFR1*, *AFR2*, *AFR3,* and *MDR1*, promote intracellular efflux of xenobiotics, attenuates the deleterious stimulatory effects of drugs on organisms, and also reduces drug sensitivity, which is one of the direct causes of *C. neoformans* presenting resistance to multiple drugs (17–19).

Prolonged clinical treatment with triazoles may trigger resistance of *C. neoformans* to triazole antifungals (20, 21). However, resistant *C. neoformans* (RCN) can also be isolated from patients who have never been treated with triazoles, such as fluconazole (22–24), suggesting that clinical medication is not the only way for patients to acquire resistance. Moreover, triazole-resistant *C. neoformans* has been recovered from fungicide-exposed plantation soil (25). Considering that triazole fungicides largely used in agriculture and medical triazole antifungal drugs are structurally similar and have the same target of action (26), the application of agricultural triazoles is likely to contribute to the development and dissemination of RCN in the environment, with the potential for patients to inhale resistant spores in the environment and thus acquire resistance (27). Although previous studies have demonstrated that certain triazole fungicides, such as tebuconazole, can induce resistance in *C. neoformans*, it remains unknown whether residual levels of triazoles in soil can promote cross-resistance to medical triazoles in *C. neoformans*. Additionally, the relationship between triazole residual levels and the frequency of RCN still needs to be explored (6, 28).

Therefore, this study utilized propiconazole as a representative triazole fungicide, which has been widely used in field disease control and wood preservation (29, 30), to investigate whether resistant *C. neoformans* could be induced by propiconazole in both liquid media and soil. The findings can enhance our knowledge about the emergence and spread of resistant *C. neoformans* in the environment, which is helpful for the prevention and treatment of cryptococcosis.

## RESULTS

### Development of triazole resistance in *C. neoformans* following exposure to propiconazole in liquid media

To determine whether propiconazole induces *C. neoformans* to develop cross-resistance to triazole drugs, six sensitive strains (NC-X-4, NC-X-7, NC-X-10, NC-X-11, NC-X-12, and P1) were subjected to propiconazole at gradually increasing concentrations, ranging from 0.125 to 16 mg/L. The MICs of isolated *C. neoformans* after exposure to propiconazole are listed in Table 1. No resistant *C. neoformans* isolate was observed from the control, suggesting that the solvent and continuous transfer process did not influence the susceptibility of *C. neoformans*. As shown in Table 1, a total of eight resistant strains (P1-1, P1-2, P1-3, NCX-7-1, NCX-7-2, NCX-10-1, NCX-11-1, and NCX-11-2) were isolated; these isolates were resistant to VOR with MIC of 1-2 mg/L. Among them, four (P1-1, P1-2, P1-3, and NCX-7-2), three (P1-1, P1-2, and P1-3), and four (P1-1, P1-2, P1-3, and NCX-7-2) strains were resistant to FLU, ITR, and POS with MICs of 16–>32, 1–2, and 0.5-1 mg/L, respectively.

**TABLE 1:** Sensitivity of tested *C. neoformans* against triazoles before and after propiconazole exposure in liquid media^*a*^

Strain no.	Strain no. after induction	Initial MIC (mg/L)					Final MIC (mg/L)				
		FLU	VOR	ITR	POS	PRO	FLU	VOR	ITR	POS	PRO
P1	P1-1	0.5	0.25	<0.0625	0.25	0.25	>32	2	1	1	8
	P1-2	0.5	0.25	<0.0625	0.25	0.25	16	1	1	1	1
	P1-3	0.5	0.25	<0.0625	0.25	0.25	32	2	2	1	8
NC-X-4	NCX-4-1	0.5	0.25	<0.0625	0.25	0.25	2	0.25	0.125	0.125	2
NC-X-7	NCX-7-1	0.5	0.25	<0.0625	0.25	0.25	8	1	0.25	0.125	1
	NCX-7-2	0.5	0.25	<0.0625	0.25	0.25	16	2	0.25	0.5	4
NC-X-10	NCX-10-1	2	0.125	<0.0625	0.0625	0.25	8	1	0.25	0.25	2
NC-X-11	NCX-11-1	2	0.125	<0.0625	0.0625	0.125	8	1	0.125	0.25	4
	NCX-11-2	2	0.125	<0.0625	0.0625	0.125	8	1	0.125	0.25	4
NC-X-12	NCX-12-1	2	0.125	<0.0625	0.0625	0.25	4	0.25	0.25	0.25	1

^*a*^ FLU, fluconazole; VOR, voriconazole; ITR, itraconazole; POS, posaconazole; PRO, propiconazole.

### Triazole resistance in *C. neoformans* induced by residual propiconazole in soil

**Fig 1:**
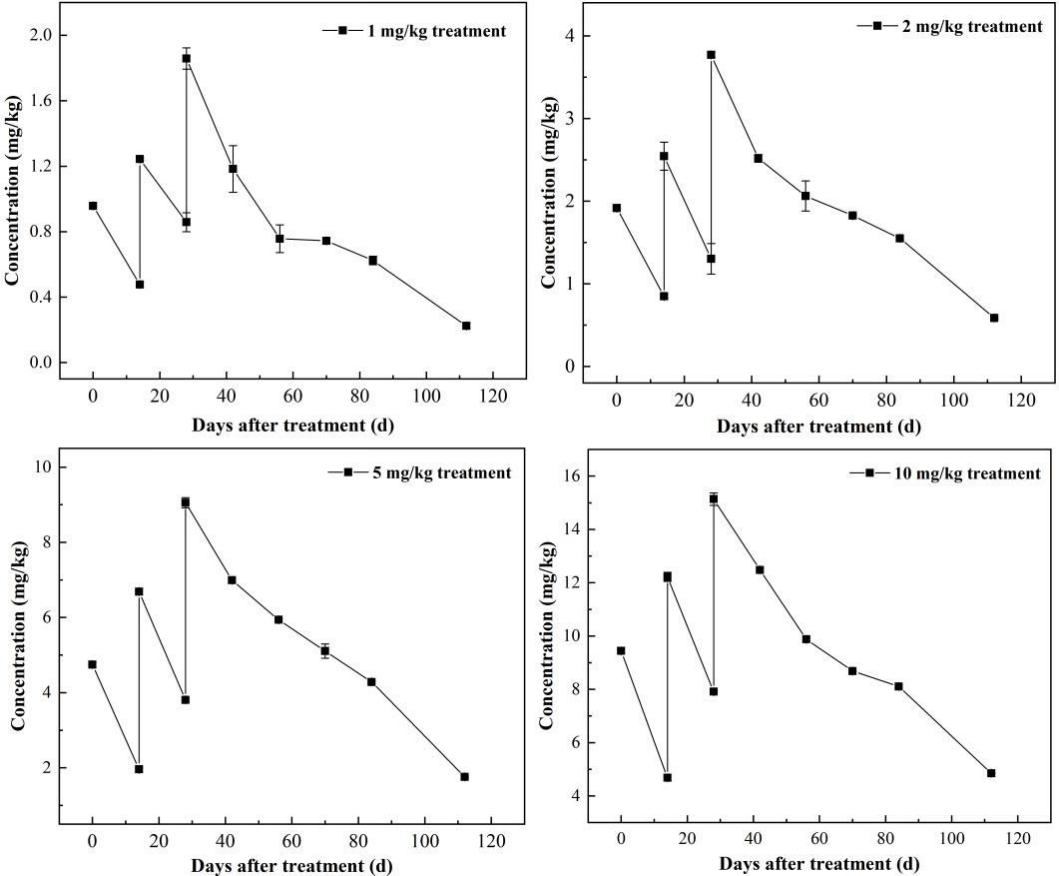
Dissipation curves of propiconazole in the soil.

To investigate whether the residues of triazole fungicides in soil can lead to the emergence of RCN and to clarify the potential relationship between the levels of fungicide residues and the resistant strains, the P1 strain labeled with hygromycin was exposed to propiconazole-treated soil. Figure 1 illustrates the dissipation pattern of propiconazole in the soil. In the treatment group with 1 mg/kg, the remaining levels of propiconazole in the soil after 14 days following the first, second, and third applications were 0.48, 0.86, and 1.18 mg/kg, respectively, indicating that continuous application leads to a persistent accumulation of propiconazole residues in the soil. Similar trends were found at 2, 5, and 10 mg/kg treatments. After the final application, the dissipation dynamics data were fitted using the first-order kinetic equation, and the half-lives were 28.9, 36.5, 43.3, and 53.3 days, and the R^2^ values of 0.944, 0.935, 0.923, and 0.985, respectively, for the treatments of 1, 2, 5, and 10 mg/kg (Table S1).

**Fig 2:**
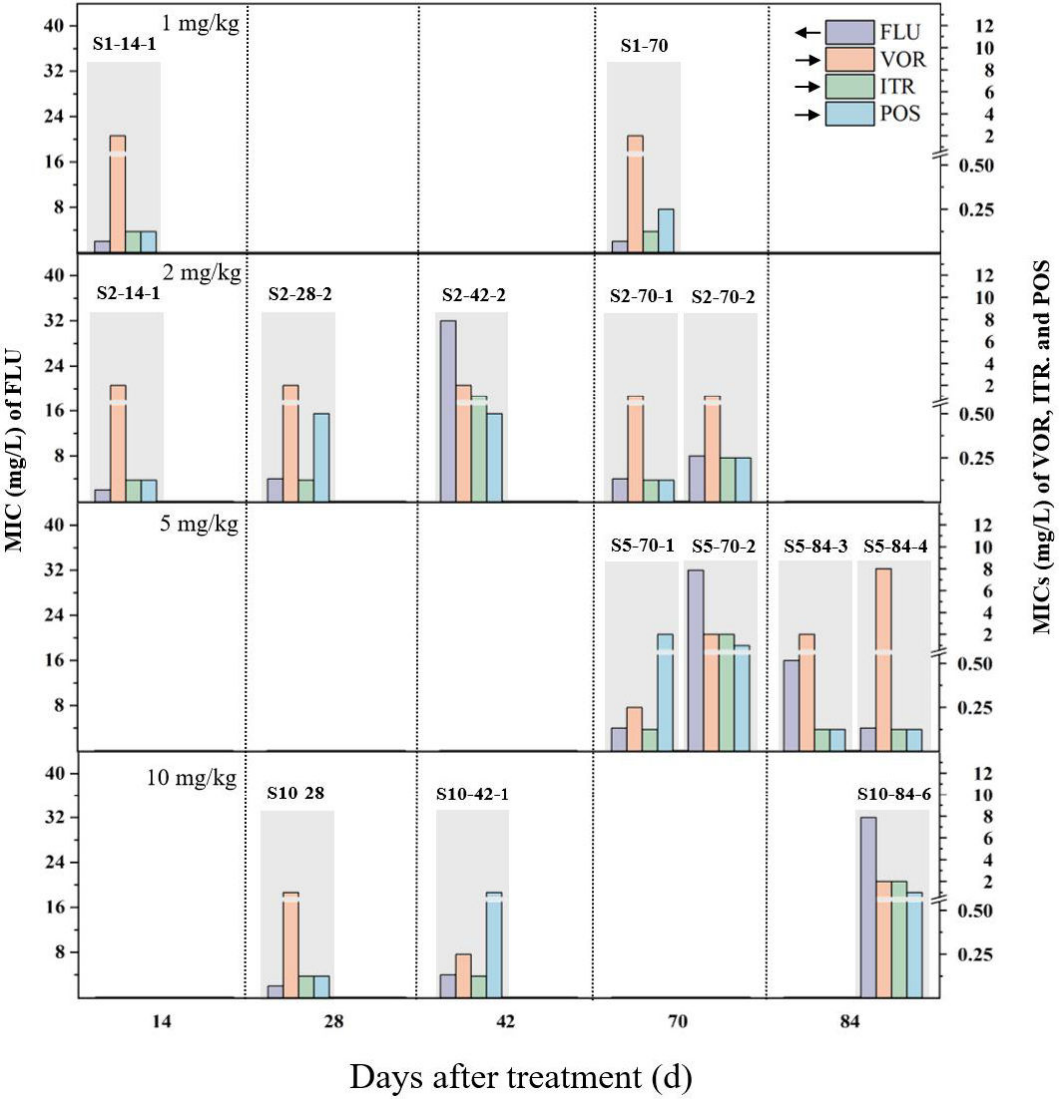
The MICs of clinical triazoles (FLU, fluconazole; VOR, voriconazole; ITR, itraconazole; POS, posaconazole) against the resistant strains of *C. neoformans* isolated from propiconazole-contaminated soil.

During this exposure period, no resistant isolates of P1 were obtained from the control. In the propiconazole-treated soils, a total of 46 strains of P1 capable of growing on the caffeic acid corn agar plates containing hygromycin, chloramphenicol, and FLU were obtained (Tables S2 to S5). Among these strains, 14 were found to exhibit resistance to medical triazoles (Fig. 2). The *HPH* gene could be amplified from these strains, suggesting that the 14 RCN isolates evolved from P1 under the selective pressure of the triazole fungicide propiconazole (Fig. S1). In the soil amended with 1 mg/kg of propiconazole, two RCN strains (S1-14-1 and S1-70) were isolated. Both strains exhibited resistance only to VOR, with an MIC of 2 mg/L. Interestingly, as the exposure concentration increased, more and more RCN isolates were obtained from propiconazole-treated soils. There were five resistant isolates of P1 (S2-14-1, S2-28-2, S2-42-2, S2-70-1, and S2-70-2) obtained from soils treated with 2 mg/kg propiconazole, four strains (S5-70-1, S5-70-2, S5-84-3, and S5-84-4) from soils treated with 5 mg/kg, and three strains (S10-28, S10-42-1, and S10-84-6) from soils treated with 10 mg/kg. Overall, 12 out of the 14 resistant strains exhibited VOR resistance, with MICs ranging from 1 to 8 mg/L. In contrast, FLU resistance and POS resistance were observed in only four and six isolates, respectively, with MICs between 16 and 32 mg/L for FLU and 0.5–2 mg/L for POS. Additionally, MIC values for ITR ranged from 1 to 2 mg/L in three isolates.

### Hereditary stability of induced strains with triazole resistance

**Fig 3:**
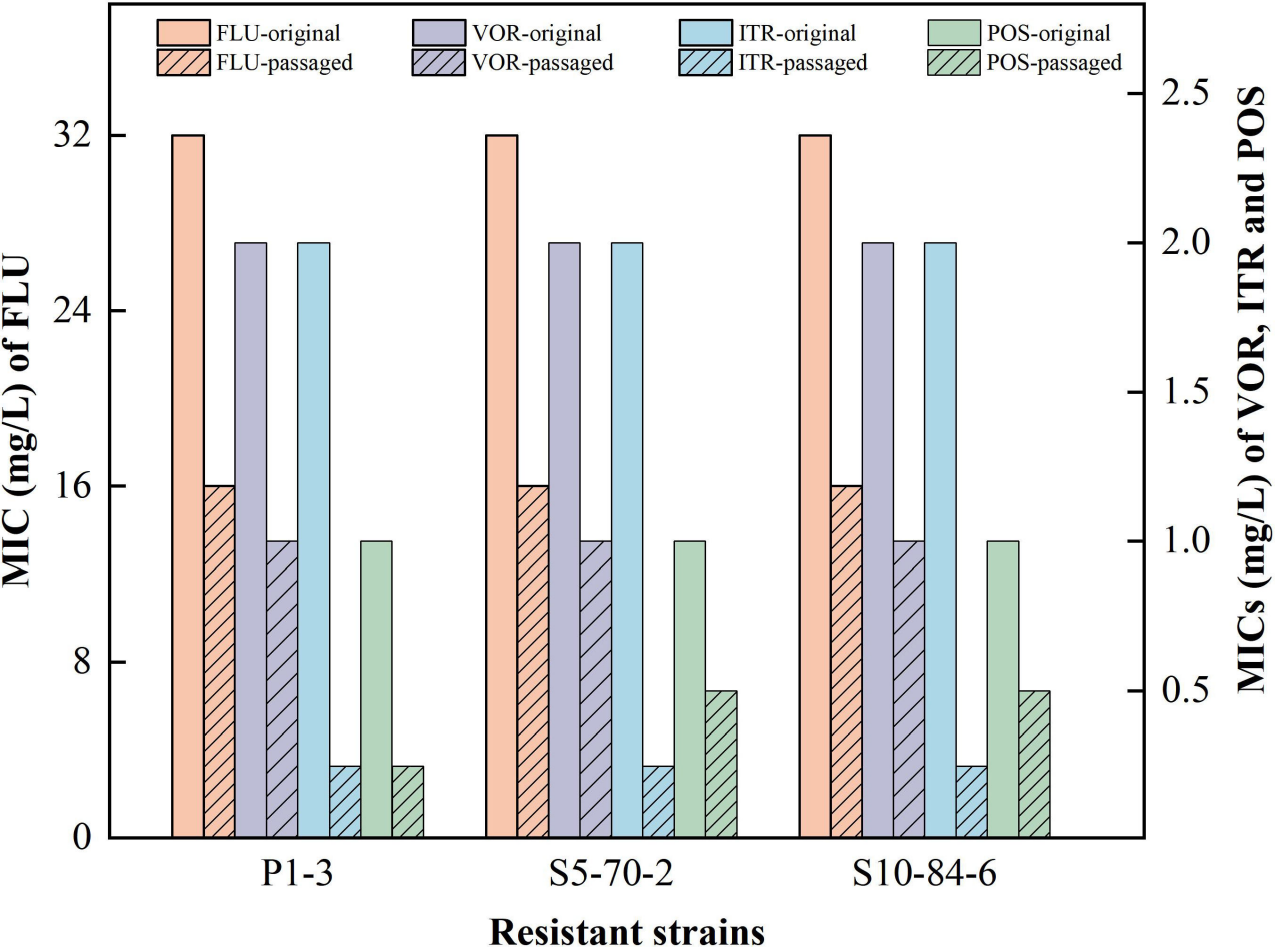
MICs of three resistant *C. neoformans* before and after 15 consecutive transfers to four medical triazoles (FLU, fluconazole; VOR, voriconazole; ITR, itraconazole; POS, posaconazole).

To assess the stability of the isolated RCN strains, a total of 22 resistant strains, which were initially isolated from liquid media and soil, were cultured on plain Sabouraud’s dextrose agar (SDA) plates. These strains were transferred every 5 days, with the process repeated for a total of 15 transfers. Table S6 shows the variations in MICs of these strains after five, ten, and fifteen transfers. As shown in Fig. 3, 3 strains (P1-3, S5-70-2, and S10-84-6) of them maintained resistance to triazole drugs. These strains continued to exhibit resistance to FLU and VOR, maintaining MICs of 16 and 1 mg/L, respectively. Furthermore, strains S5-70-2 and S10-84-6 also demonstrated resistance to POS, with an MIC of 0.5 mg/L. In contrast, the remaining strains regained susceptibility to medical triazoles following 15 consecutive transfers.

### Potential mechanism of resistant *C. neoformans* strains

**Fig 4:**
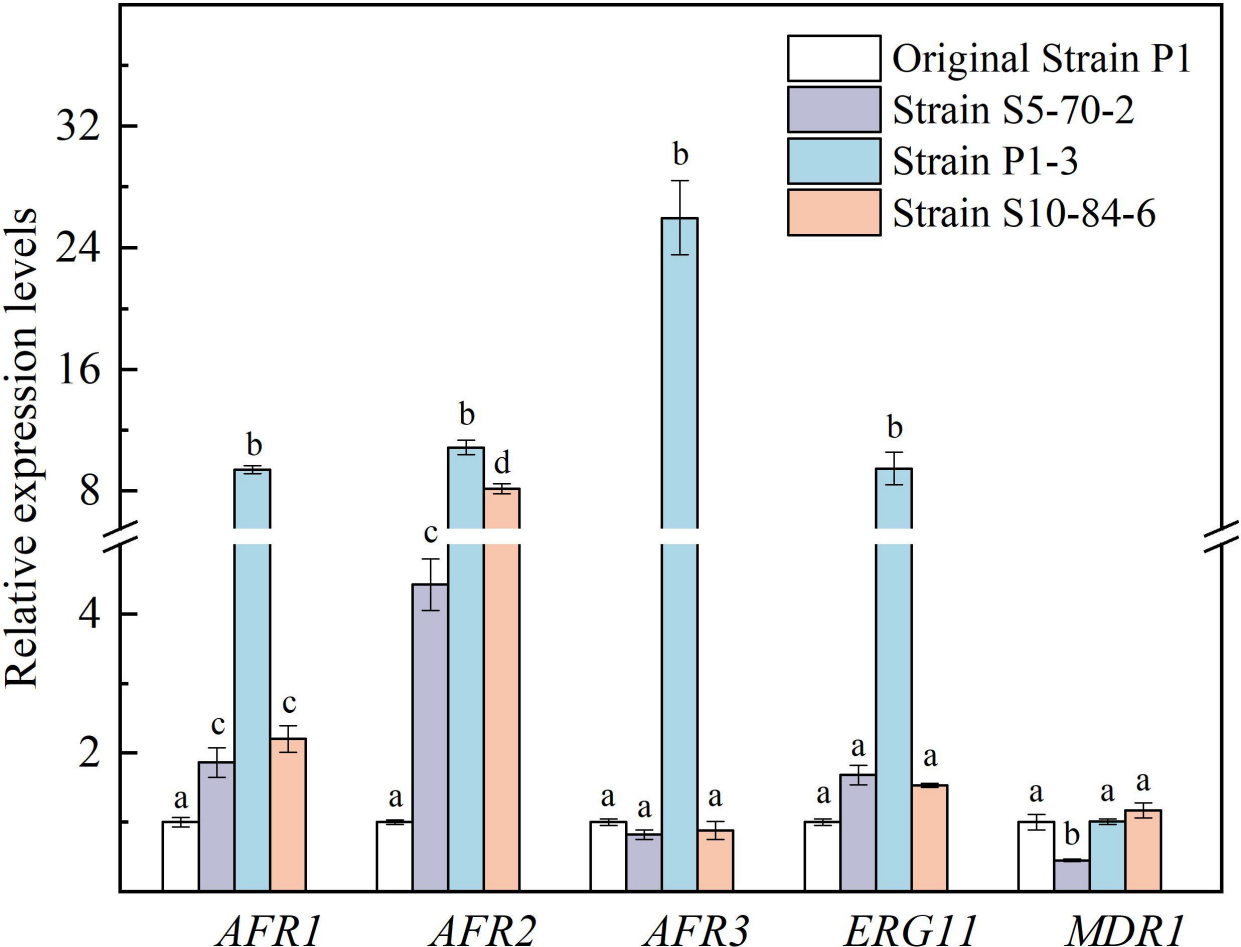
Relative expression levels of *ERG11*, *AFR1*, *AFR2*, *AFR3*, and *MDR1* of the three resistant strains with stable resistance inheritance. Expression levels of *ERG11* and these efflux pump genes were evaluated in comparison with the expression of these same genes in the original strain P1, in the absence of the pesticide used to obtain the tested isolates. The letters indicate the difference in expression levels (*P* < 0.05, Duncan’s test).

To investigate potential mutations, the *ERG11* gene of the three resistance-stable strains was sequenced and analyzed. No mutations were found in the target gene of any of these strains. Given that efflux pumps significantly contribute to the resistance of *C. neoformans* to triazole drugs, the transcription levels of the *ERG11* gene and key efflux pump genes (*AFR1*, *AFR2*, *AFR3*, and *MDR1*) were determined using qRT-PCR. As shown in Fig. 4, the transcription levels of *AFR1* and *AFR2* in isolates P1-3, S5-70-2, and S10-84-6 were 1.86–9.40- and 4.42–10.86-fold (*P* < 0.05) increases over their original strain P1, respectively. The *ERG11* and *AFR3* levels in strain P1-3 were 9.48 and 25.97 times (*P* < 0.05) higher than strain P1, respectively.

## DISCUSSION

**Fig 5:**
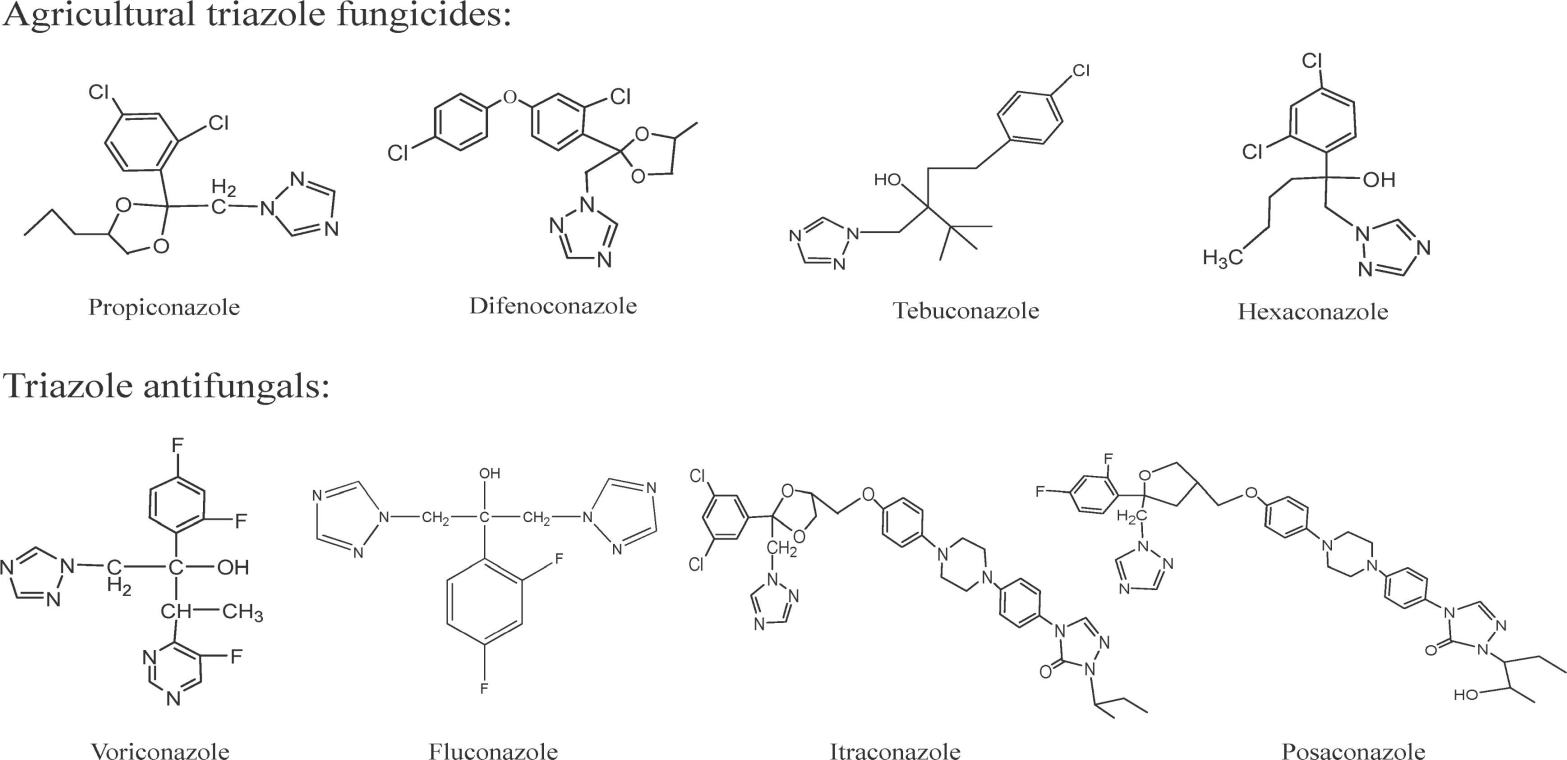
The chemical structures of propiconazole, its major competitors, and triazole antifungals.

The intensive use of triazole fungicides in agriculture represents a potential environmental selection pressure for triazole resistance in *Cryptococcus*. Propiconazole and its major competitors (e.g., tebuconazole, difenoconazole, hexaconazole) are extensively used globally to control a broad spectrum of fungal diseases in cereals (wheat, barley, rice), turf grasses, fruit trees (e.g., against rusts, powdery mildews, leaf spots), and other crops (26, 28). Structurally (Fig. 5), propiconazole, tebuconazole, and hexaconazole are classified as “conazole-type” triazoles, bearing a closer resemblance to FLU. This class is characterized by two or three triazole rings attached via an ether linkage to a central core, often with chlorinated phenyl rings. In contrast, triazoles like difenoconazole possess more complex fused ring systems, exhibiting structural features intermediate between FLU and ITR derivatives. The intensive application of FLU-like conazoles, particularly propiconazole, near environmental niches of *Cryptococcus* suggests they may play a role in selecting for resistance mechanisms relevant to human health, including heteroresistance. Our study provides direct evidence that propiconazole, a persistent agricultural triazole, induces resistance in *C. neoformans* through dual mechanisms of *ERG11* overexpression and efflux pump activation. Crucially, this induction occurs even at field-relevant concentrations in soil (1–10 mg/kg).

Triazole-resistant *C. neoformans* strains are increasingly documented in both environmental and clinical settings (22, 24, 25, 31–33). However, the role of agricultural triazole fungicides in driving such resistance is not well understood. While studies on *Aspergillus* spp. dominate this field (15, 26, 30, 34, 35), our data and recent reports implicate agricultural triazoles as potential drivers of cross-resistance in cryptococci. For instance, tebuconazole exposure induces cross-resistance to clinical triazoles (FLU, ITR) in *C. neoformans* and *C. gattii* (6), and short-term (5–7 days) exposure to common agricultural triazoles reduces FLU susceptibility and promotes multidrug-resistant phenotypes (28). Notably, in our 56-day propiconazole adaptation experiment, we generated eight resistant strains exhibiting significantly elevated MICs to FLU (16–>32 mg/L), VOR (1–2 mg/L), ITR (1–2 mg/L), and POS (0.5–1 mg/L). This propensity for FLU resistance was stronger than previously reported (28), aligning with intrinsic antifungal efficacy differences: ITR and POS exhibit superior activity against *C. neoformans*, whereas FLU shows weaker inhibition (36, 37). These findings position propiconazole as a high-risk fungicide for cryptococcal cross-resistance.

Direct evidence linking the emergence of RCN to triazole fungicide residues in environmental samples like soil remains limited, and whether typical soil residues can select for resistant *C. neoformans* is controversial. Takahashi et al. (25) recovered a non-wild-type *C. neoformans* strain from fungicide-treated soil (FLU MIC = 32 mg/L). However, critics argue that typical soil residue levels (0.5–2 mg/kg) may be insufficient to induce resistance compared to therapeutic concentrations (up to 11 mg/L in serum) (38–40). To address this, we simulated field-relevant propiconazole exposures (1–10 mg/kg) and observed a clear concentration-dependent increase in resistant strains: treatments of 1, 2, 5, 10 mg/kg yielded two, five, four, and three resistant isolates, respectively, with corresponding measured exposure levels of 0.22–1.86, 0.59–3.77, 1.76–9.05, and 4.68–15.13 mg/kg. These results mirror findings in *A. fumigatus*, where propiconazole residues correlate with azole-resistant strain prevalence (41, 42). Importantly, our soil model confirms that although propiconazole residues in soil (0.22–15.13 mg/kg) are substantially lower than therapeutic serum concentrations (11 mg/L), their localized enrichment at the soil-microbial interface may exert comparable selective pressure. This pressure favors resistant clones through inhibitory rather than fungicidal effects (43).

Classical models attribute triazole resistance in *C. neoformans* primarily to non-synonymous mutations in the target gene *ERG11* (10, 11, 19). However, analysis of our resistant strains revealed no *ERG11* mutations, echoing reports by Drakulovski et al. (28) where fluconazole-adapted strains also retained wild-type *ERG11*. Alternative mechanisms include heteroresistance, efflux pump upregulation, or *ERG11* overexpression (13, 14, 17). Heteroresistance is a transient phenotypic adaptation: sustained drug pressure selectively enriches adaptive subpopulations, elevating the population-level MIC; subsequent pressure removal permits reversion to susceptibility (14). Critically, environmental exposure to sublethal propiconazole concentrations may impose persistent selection pressure on *Cryptococcus* populations. Our data show that such pressure amplifies the proportion of strains exhibiting transient fluconazole resistance through preferential expansion of heteroresistant subpopulations. In resistance induction assays, triazole resistance emerged in all 20 propiconazole-exposed strains. After 15 generations of drug-free subculture, susceptibility was restored in 19 strains. However, three strains (13.6%) retained stable resistance phenotypes. This sustained resistance, which persists in the absence of drug pressure, stands in contrast to the transient nature of heteroresistance and indicates the induction of stable genetic or epigenetic alterations. These findings establish that environmentally relevant propiconazole exposures not only select for pre-existing resistant subclones but actively drive *de novo* induction of drug resistance in *C. neoformans*.

To dissect these mechanisms, we quantified the expression of *ERG11* and four efflux pump genes (*AFR1*, *AFR2*, *AFR3*, *MDR1*) in propiconazole-adapted strains P1-3, S5-70-2, S10-84-6. Two distinct yet complementary pathways emerged: (i) ubiquitous efflux activation: all strains exhibited pronounced upregulation of *AFR1* (1.86–9.40-fold, *P* < 0.05) and *AFR2* (4.42–10.86-fold, *P* < 0.05), identifying these pumps as a conserved mechanism for triazole extrusion; (ii) divergent secondary adaptations: strain P1-3 uniquely overexpressed *ERG11* (9.48-fold) alongside an extraordinary *AFR3* induction (25.97-fold), suggesting synergistic target-enhancement and niche-specific efflux in soil-adapted clones. This mechanistic plasticity mirrors observations in tebuconazole-exposed *C. neoformans* (6). However, propiconazole uniquely mobilizes *AFR3*, an understudied efflux pump previously not reported in agricultural triazole resistance. We propose that environmental triazoles drive clinical cross-resistance through a hierarchical adaptation process: constitutive efflux via *AFR1*/*AFR2* establishes baseline tolerance, while stochastic induction of *ERG11* or *AFR3* enables subpopulations to withstand higher drug pressures. Future investigations should prioritize identifying transcriptional regulators (e.g., SltA) that orchestrate this adaptive hierarchy and understanding how propiconazole specifically triggers the *de novo* induction pathway, distinct from simple selection, particularly within soil reservoirs where residual propiconazole sustains selective pressure (44).

Our study bridges a critical gap between agricultural fungicide use and clinical cryptococcosis outcomes. Propiconazole-induced RCN strains could disseminate via aerosolized soil or food chains (45, 46), potentially compromising triazole therapies. To mitigate this risk, we recommend integrating soil resistance surveillance into triazole fungicide usage guidelines and implementing strategies such as crop rotation or alternative fungicides to reduce propiconazole accumulation. However, our laboratory model simplifies real-world conditions by omitting microbial competition and environmental stressors (e.g., thermal fluctuations). Field validation is needed to confirm resistance persistence in complex soil ecosystems. This approach is crucial for understanding RCN strain evolution and developing prevention strategies from a fungicide management perspective.

## MATERIALS AND METHODS

### Chemicals and strains

FLU (>98%), ITR (>98%) and POS (>99.9%) were obtained from Sigma-Aldrich Co., USA. VOR (>99.9%) and propiconazole (>99.0%) were obtained from Dr. Ehrenstorfer GmbH, Germany. Other chemicals used in this study were analytically pure.

Six wild-type strains (NC-X-4, NC-X-7, NC-X-10, NC-X-11, NC-X-12, and P1) of *C. neoformans* were used to observe their changes in susceptibility to triazole drugs after sequential exposure to propiconazole. The strains NC-X-4, NC-X-7, NC-X-10, NC-X-11, and NC-X-12 were isolated from environment soils in Nanchang, Jiangxi, China. Strain P1 was derived from strain NC-X-7 by introducing the hygromycin resistance gene. The transformation method is described in the Supporting Information. The minimal inhibitory concentrations (MICs) of these strains against triazoles are listed in Table 1.

### Resistance induction of *C. neoformans* by propiconazole in liquid media

The strains of *C. neoformans* were plated on Sabouraud’s dextrose agar (SDA) plates and cultured at 35°C for 3 days. Then, the colonies were dissolved in 1 mL of 0.9% NaCl solution and diluted with 0.2× Sabouraud’s dextrose broth (SDB) medium to 4 × 10^6^ CFU/mL. After initial mixing of 0.5 mL suspension with 1.5 mL 0.2× SDB medium (supplemented with 0.125 mg/L PRO for strains P1/NC-X-4/7/10/12, or 0.0625 mg/L PRO for NC-X-11), cultures were incubated at 35°C for 7 days. Subsequently, 0.5 mL aliquots were transferred to fresh medium containing doubled PRO concentrations (0.25 and 0.125 mg/L respectively) for continued cultivation. The transfer process was repeated once a week for a total of seven transfers, during which the PRO concentration was gradually increased to 16 mg/L. The *C. neoformans* strains cultured in 0.2× SDB medium without exposure to PRO served as controls. All experiments were performed in triplicate.

### Influence of residual propiconazole in soil on resistance of *C. neoformans*

Topsoil samples (0–10 cm depth) were obtained from Jiangxi Agricultural University, Nanchang, China, and the propiconazole residues were below the limit of detection. Soil samples were dried naturally and sieved through a 2-mm sieve for subsequent experiments. The physicochemical properties of the soil were detected and listed as follows: pH, 4.91; organic matter content, 29.4 g/kg; total N, 0.180%; total P, 0.039%; cation exchange capacity, 10.2 cmol/kg; clay, 18.38%; sand, 53.03%; silt, 28.59%.

The soil samples (100 g, equal to its dry weight) were mixed with propiconazole in acetone. After volatilization of acetone, the treated soils were mixed with the remaining soil sample (400 g) to produce final concentrations of 1, 2, 5, and 10 mg/kg of propiconazole, respectively, corresponding to 1, 2, 5, and 10 times the initial field deposition (28, 38). Soil added with equal amounts of acetone was considered as the control. Subsequently, appropriate amounts of deionized water and P1 strain solution were added so that the water content of the soil was 60% of the maximum water holding capacity of the field and the final concentration of P1 in the soil was 10^7^ CFU/g. The soil was passed through a 2-mm sieve and then placed into a plastic pot (130 mm × 115 mm × 88 mm). The pots were covered with tin foil and incubated at 25°C. Two additional amendments of propiconazole were conducted at 14-day intervals. Soil samples (20 g) were collected on days 0, 14, and 28 after the first two treatments, as well as on days 0, 14, 28, 42, 56, and 84 following the third application, to isolate RCN and determine propiconazole residue. Each treatment was completed in triplicate.

### Isolation and identification of RCN strains from liquid media and soil

Following incubation in liquid medium, 100 µL cultures were plated on SDA supplemented with FLU (16 mg/L) and screened for RCN strains through 5-day dark incubation at 35°C.

Following soil incubation, propiconazole-treated soil samples (3–5 g) were homogenized in 30 mL of 0.9% NaCl solution and shaken at 150 rpm (25°C) for 2 h. Then, 100 µL supernatant was spread on the caffeic acid corn agar plates containing hygromycin (100 mg/L), chloramphenicol (100 mg/L), and FLU (16 mg/L). After a 5-day incubation at 35°C, suspected P1 isolates were selected, purified, and identified via morphological analysis combined with ITS sequencing and hygromycin resistance gene amplification (1, 25). Primer sequences are provided in Table S7.

### Determination of susceptibility of *C. neoformans*

The susceptibility test of *C. neoformans* strains against triazole drugs (FLU, ITR, POS, and VOR) was conducted according to the broth dilution method recommended by CLSI M27-A4. Briefly, the triazole compounds were gradient dilution using RPMI 1640 medium containing 2% glucose in a 96-well plate. The *C. neoformans* strains were collected and diluted into a concentration of 5 × 10^3^ CFU/mL. Second, 100 µL of strain solution was mixed with triazole solutions, and the prepared plates were sealed and then maintained at 35°C for 72 h. The minimum inhibitory concentration (MIC) is defined as the lowest concentration of the drug that inhibits 50% of growth compared to drug-free controls. *Candida krusei* ATCC 6258 and *Candida parapsilosis* ATCC 22019 were used as the quality control strains, and the MIC test for each strain was conducted in triplicate. According to the epidemiological cut-off values, MIC >8 mg/L for FLU, and MIC >0.25 mg/L for ITR, VOR, and POS, were defined as resistant strains, respectively (47).

### Susceptibility hereditary

To determine whether resistance to triazole drugs in *Cryptococcus neoformans* can be stably inherited, *C. neoformans* strains with resistant phenotypes were inoculated in blank SDA plates and transferred every 5 days for 15 consecutive generations. The sensitivity of *C. neoformans* strains to triazole drugs was tested every five transfers, as described above.

### Detection of *ERG11* gene mutation

DNA of resistant *C. neoformans* isolates was extracted by the Ezup Column Yeast Genomic DNA Purification Kit (Sangon Biotech, Shanghai, China). The *ERG11* gene was amplified through the primers of *erg11*-HF3 (5′GTACATACCCGCTAGCTTACTCTCG3′) and *erg11*-HR3 (5′GCCTATGACTTCATGACCTCTTTC3′). The sequence was compared with that of the susceptible *C. neoformans* strain (GenBank No. AY376725).

### Determination of *ERG11* and efflux pump gene expression levels

Strains that remained resistant after 15 transitions were cultured on blank SDA plates, and total RNA was extracted using the FlashPure Yeast Total RNA Mini Kit (GeneBetter, Beijing, China) according to the instructions. The expression levels of the target gene (*ERG11*) and four efflux pump genes (*AFR1*, *AFR2*, *AFR3*, *MDR1*) were determined by qRT-PCR according to Drakulovski et al. (28). The *ACTIN1* was used as the internal control. The related primers are listed in Table S7. The expression of each gene for each strain was tested in triplicate. The 2^-△△CT^ method was utilized to analyze the qRT-PCR data (48).

### Analysis of propiconazole residues in soil

Propiconazole was extracted from soil using the QuEChERS method as reported by Cao et al. (30), with slight adjustments. Totally, approximately 5 g of soil samples (dry weight) were mixed with 20 mL of an acetonitrile-water solution (v/v: 1:1) and subjected to ultrasound for 20 min. Following that, 4 g of anhydrous MgSO_4_ and 1 g of NaCl were introduced to the vessel, which was then vortexed for 30 s and centrifuged for 5 min. After centrifugation, the supernatant (1.5 mL) was filtered and prepared for HPLC measurement. The residue concentration of propiconazole was detected using an Agilent 1260 HPLC coupled with a reverse-phase C18 column (4.6 mm × 250 mm, 5 µm particle size) maintained at 30°C. Detection was performed at 220 nm using a DAD detector. The mobile phase was composed of a mixture of acetonitrile and water (70:30, v/v) with a constant rate of 1 mL/min.

## SUPPLEMENTAL MATERIAL

Supplemental material aac.00765-25-s0001.docx Tables S1 to S7; Fig. S1.

ASM does not own the copyrights to Supplemental Material that may be linked to, or accessed through, an article. The authors have granted ASM a non-exclusive, world-wide license to publish the Supplemental Material files. Please contact the corresponding author directly for reuse.
