# Supplementary material for: Agricultural propiconazole residues promote triazole cross-resistance in Cryptococcus neoformans through ERG11 and efflux pump overexpression
Source: Antimicrob Agents Chemother. 2025 Jul 31;69(9):e00765-25. doi: 10.1128/aac.00765-25 (PMC12406658; doi:10.1128/aac.00765-25)
Supplement: Supplemental material — Tables S1 to S7; Fig. S1. [file aac.00765-25-s0001.docx]

**Supporting Information**

**Agricultural propiconazole residues promote triazole cross-resistance in *Cryptococcus neoformans* through *ERG11* and efflux pump overexpression**

Hantao Yu^a,1^, Wenwen Peng^a,b,1^, Hang Li^a^, Ying Zhang^a^, Xiaoxiang Fu^a,b^, Xia Gong^c^, Hongyi Wei^a^, Qinghong Zhou^b^, Yingjin Huang^b^, Duantao Cao^a,b,*^

^a^ The Laboratory for Phytochemistry and Botanical Pesticides, College of Agriculture, Jiangxi Agricultural University, Nanchang 330045, China; ^b^ Jiangxi Province Key Laboratory of Vegetable Cultivation and Utilization (Jiangxi Agricultural University), Nanchang 330045, China; ^c^ College of Chemistry and Materials, Jiangxi Agricultural University, Nanchang 330045, China.

**Running title: Propiconazole induce triazole resistance in *Cryptococcus neoformans***

^1^ These authors contributed equally to this work.

*Corresponding author.

Tel/Fax: +86-791-83813185

E-mail address: [cdtagri@jxau.edu.cn](mailto:cdtagri@jxau.edu.cn)

The method for constructing the hygromycin-labeled strain P1 was as follows: the pSM565 plasmid was first linearized by BamHI enzyme and then transformated into the susceptible recipient strain NC-X-12 using the protocol described by Pan et al. (2002). Transformant P1 was selected on Sabouraud’s dextrose agar (SDA) plate supplemented with 100 mg/L of hygromycin B and verified by amplifying the hygromycin gene.

**Reference**

Pan WH, Liao WQ, Gu JL, Huo KK. 2002. Highly effective transformation of *Cryptococcus neoformans*. Acad J Sec Mil Med Univ 23: 1218–1220.

Table S1 Kinetic data of propiconazole dissipation in soil.

| Propiconazole concentration (mg/kg) | Kinetic equation | DT_50_ (d) | R^2^ |
| --- | --- | --- | --- |
| 1 | $C=3.431e^{-0.024t}$ | 28.9 | 0.944 |
| 2 | $C=6.627e^{-0.019t}$ | 36.5 | 0.935 |
| 5 | $C=14.780e^{-0.016t}$ | 43.3 | 0.923 |
| 10 | $C=21.934e^{-0.013t}$ | 53.3 | 0.985 |

DT_50_: degradation half-lives

Table S2 MICs of *C. neoformans* isolated from soils treated with propiconazole at 1 mg/kg.

| Exposure time (d) | Strain No. | MICs (mg/L) for Triazole drugs | | | | |
| --- | --- | --- | --- | --- | --- | --- |
|  |  | FLU | VOR | ITR | POS | PRO |
| 14 | S1-14-1 | 2 | 2 | < 0.125 | 0.125 | 0.5 |
|  | S1-14-2 | 2 | 0.25 | 0.125 | < 0.125 | 0.5 |
|  | S1-14-3 | 2 | 0.25 | < 0.125 | 0.125 | 0.5 |
|  | S1-14-4 | 2 | 0.25 | 0.125 | 0.125 | 0.5 |
| 42 | S1-42 | 1 | 0.25 | 0.125 | 0.25 | 0.25 |
| 70 | S1-70 | 2 | 2 | < 0.125 | 0.25 | 16 |
| 84 | S1-84 | 1 | 0.125 | 0.125 | 0.125 | 0.125 |

FLU, fluconazole; VOR, voriconazole; ITR, itraconazole; POS, posaconazole; PRO, propiconazole.

Table S3 MICs of *C. neoformans* isolated from soils treated with propiconazole at 2 mg/kg.

| Exposure time (d) | Strain No. | MICs (mg/L) for Triazole drugs | | | | |
| --- | --- | --- | --- | --- | --- | --- |
|  |  | FLU | VOR | ITR | POS | PRO |
| 14 | S2-14-1 | 2 | 2 | < 0.125 | 0.125 | 0.25 |
|  | S2-14-2 | 2 | 0.25 | < 0.125 | <0.125 | 0.5 |
|  | S2-14-3 | 2 | 0.25 | < 0.125 | < 0.125 | 0.5 |
| 28 | S2-28-1 | 1 | 0.25 | < 0.125 | < 0.125 | < 0.125 |
|  | S2-28-2 | 4 | 2 | 0.125 | 0.5 | 4 |
| 42 | S2-42-1 | 2 | 0.25 | < 0.125 | 0.25 | 0.25 |
|  | S2-42-2 | 32 | 2 | 1 | 0.5 | 8 |
| 70 | S2-70-1 | 4 | 1 | 0.125 | 0.125 | 2 |
|  | S2-70-2 | 8 | 1 | 0.25 | 0.25 | 8 |
| 84 | S2-84 | 2 | 0.125 | 0.125 | 0.125 | 0.25 |
| 112 | S2-112 | 2 | 0.25 | 0.125 | 0.125 | 0.125 |

FLU, fluconazole; VOR, voriconazole; ITR, itraconazole; POS, posaconazole; PRO, propiconazole.

Table S4 MICs of *C. neoformans* isolated from soils treated with propiconazole at 5 mg/kg.

| Exposure time (d) | Strain No. | MICs (mg/L) for Triazole drugs | | | | |
| --- | --- | --- | --- | --- | --- | --- |
|  |  | FLU | VOR | ITR | POS | PRO |
| 28 | S5-28-1 | 4 | 0.25 | < 0.125 | 0.125 | 0.125 |
|  | S5-28-2 | 2 | 0.25 | < 0.125 | < 0.125 | < 0.125 |
| 56 | S5-56-1 | 1 | < 0.125 | < 0.125 | < 0.125 | 0.25 |
|  | S5-56-2 | 1 | < 0.125 | < 0.125 | < 0.125 | 0.25 |
| 70 | S5-70-1 | 4 | 0.25 | 0.125 | 2 | 0.25 |
|  | S5-70-2 | 32 | 2 | 2 | 1 | 16 |
| 84 | S5-84-1 | 1 | 0.125 | 0.125 | 0.125 | 0.125 |
|  | S5-84-2 | 2 | 0.25 | 0.125 | 0.125 | 0.25 |
|  | S5-84-3 | 16 | 2 | 0.125 | 0.125 | 0.25 |
|  | S5-84-4 | 4 | 8 | 0.125 | 0.125 | 0.25 |
|  | S5-84-5 | 2 | 0.125 | 0.125 | 0.125 | 0.25 |

FLU, fluconazole; VOR, voriconazole; ITR, itraconazole; POS, posaconazole; PRO, propiconazole.

Table S5 MICs of *C. neoformans* isolated from soils treated with propiconazole at 10 mg/kg.

| Exposure time (d) | Strain No. | MICs (mg/L) for Triazole drugs | | | | |
| --- | --- | --- | --- | --- | --- | --- |
|  |  | FLU | VOR | ITR | POS | PRO |
| 14 | S10-14-1 | 2 | 0.25 | 0.125 | < 0.125 | 0.25 |
|  | S10-14-2 | 2 | 0.25 | 0.125 | < 0.125 | 0.5 |
| 28 | S10-28 | 2 | 1 | 0.125 | 0.125 | 0.5 |
| 42 | S10-42-1 | 4 | 0.25 | 0.125 | 1 | 0.5 |
|  | S10-42-2 | 4 | 0.25 | < 0.125 | 0.25 | 0.25 |
| 56 | S10-56-1 | 1 | < 0.125 | < 0.125 | < 0.125 | 0.25 |
|  | S10-56-2 | 1 | < 0.125 | < 0.125 | < 0.125 | 0.25 |
|  | S10-56-3 | 1 | < 0.125 | < 0.125 | < 0.125 | 0.25 |
| 70 | S10-70-1 | 4 | 0.25 | 0.125 | 0.25 | 4 |
|  | S10-70-2 | 2 | 0.125 | 0.25 | 0.125 | 1 |
|  | S10-70-3 | 2 | 0.125 | 0.25 | 0.125 | 1 |
| 84 | S10-84-1 | 1 | 0.25 | 0.125 | 0.125 | 0.25 |
|  | S10-84-2 | 2 | 0.25 | 0.25 | 0.125 | 0.125 |
|  | S10-84-3 | 1 | 0.125 | 0.125 | 0.125 | 0.125 |
|  | S10-84-4 | 2 | 0.25 | 0.125 | 0.125 | 0.25 |
|  | S10-84-5 | 2 | 0.25 | 0.125 | 0.125 | 0.25 |
|  | S10-84-6 | 32 | 2 | 2 | 1 | 16 |

FLU, fluconazole; VOR, voriconazole; ITR, itraconazole; POS, posaconazole; PRO, propiconazole.

Table S6 Changes in the sensitivity of resistant *C. neoformans* after 15 consecutive transfers.

| Strain No. | MICs after  induced (mg/L) | | | | MICs after  five transformation (mg/L) | | | | MICs after  ten transformation (mg/L) | | | | MICs after  fifteen transformation (mg/L) | | | |
| --- | --- | --- | --- | --- | --- | --- | --- | --- | --- | --- | --- | --- | --- | --- | --- | --- |
|  | FLU | VOR | ITR | POS | FLU | VOR | ITR | POS | FLU | VOR | ITR | POS | FLU | VOR | ITR | POS |
| P1-1 | > 32 | 2 | 1 | 1 | 8 | 1 | 0.125 | 0.25 | 4 | 0.5 | 0.125 | 0.5 | 2 | 0.25 | 0.125 | 0.25 |
| P1-2 | 16 | 1 | 1 | 1 | 4 | 0.25 | < 0.125 | 0.25 | 2 | 0.25 | < 0.125 | 0.25 | 2 | 0.25 | <0.125 | 0.25 |
| **P1-3** | **32** | **2** | **2** | **1** | **32** | **2** | **0.5** | **0.5** | **16** | **1** | **0.5** | **0.5** | **16** | **1** | **0.25** | **0.25** |
| NCX-10-1 | 8 | 1 | 0.25 | 0.25 | 8 | 1 | 0.25 | 0.25 | 8 | 0.5 | 0.125 | 0.125 | 4 | 0.25 | 0.125 | 0.125 |
| NCX-11-1 | 8 | 1 | 0.125 | 0.25 | 8 | 0.25 | 0.125 | 0.25 | 4 | 0.25 | 0.125 | 0.125 | 4 | 0.25 | 0.125 | 0.125 |
| NCX-11-2 | 8 | 1 | 0.125 | 0.25 | 4 | 1 | 0.125 | 0.125 | 4 | 0.25 | < 0.125 | 0.125 | 4 | 0.25 | < 0.125 | 0.125 |
| NCX-7-1 | 8 | 1 | 0.25 | 0.125 | 8 | 0.25 | 0.125 | 0.125 | 8 | 0.25 | 0.125 | 0.125 | 8 | 0.25 | 0.125 | 0.125 |
| NCX-7-2 | 16 | 2 | 0.25 | 0.5 | 16 | 0.5 | 0.25 | 0.25 | 4 | 0.25 | 0.125 | 0.125 | 4 | 0.25 | 0.125 | 0.125 |
| S1-14-1 | 2 | 2 | < 0.125 | 0.125 | 0.5 | 0.25 | < 0.125 | 0.25 | 0.5 | 0.25 | < 0.125 | 0.125 | 0.5 | 0.25 | < 0.125 | 0.125 |
| S1-70 | 2 | 2 | < 0.125 | 0.25 | 2 | 0.5 | < 0.125 | 0.125 | 1 | < 0.125 | < 0.125 | 0.125 | 1 | <0.125 | < 0.125 | 0.125 |
| S2-14-1 | 2 | 2 | < 0.125 | 0.125 | 0.5 | 0.25 | < 0.125 | 0.25 | 0.5 | 0.25 | < 0.125 | 0.25 | 0.5 | 0.25 | < 0.125 | 0.25 |
| S2-28-2 | 4 | 2 | 0.125 | 0.5 | 4 | 0.5 | 0.125 | 0.125 | 2 | 0.5 | < 0.125 | 0.125 | 0.5 | 0.125 | < 0.125 | 0.125 |

Table S6 Continued.

| Strain No. | MICs after  induced (mg/L) | | | | MICs after  five transformation (mg/L) | | | | MICs after  ten transformation (mg/L) | | | | MICs after  fifteen transformation (mg/L) | | | |
| --- | --- | --- | --- | --- | --- | --- | --- | --- | --- | --- | --- | --- | --- | --- | --- | --- |
|  | FLU | VOR | ITR | POS | FLU | VOR | ITR | POS | FLU | VOR | ITR | POS | FLU | VOR | ITR | POS |
| S2-42-2 | 32 | 2 | 1 | 0.5 | 16 | 1 | 0.125 | 0.5 | 8 | 0.5 | 0.125 | 0.25 | 2 | 0.25 | 0.125 | 0.25 |
| S2-70-1 | 4 | 1 | 0.125 | 0.125 | 4 | 0.5 | < 0.125 | 0.125 | 2 | 0.5 | < 0.125 | 0.125 | 0.5 | 0.25 | < 0.125 | 0.125 |
| S2-70-2 | 8 | 1 | 0.25 | 0.25 | 4 | 0.5 | < 0.125 | 0.125 | 1 | 0.25 | < 0.125 | 0.25 | 0.5 | 0.25 | < 0.125 | 0.25 |
| S5-70-1 | 4 | 0.25 | 0.125 | 2 | 0.5 | 0.25 | < 0.125 | 0.25 | 0.5 | 0.25 | < 0.125 | 0.25 | 0.5 | 0.25 | < 0.125 | 0.25 |
| **S5-70-2** | **32** | **2** | **2** | **1** | **32** | **2** | **0.5** | **0.5** | **16** | **1** | **0.5** | **0.5** | **16** | **1** | **0.25** | **0.5** |
| S5-84-3 | 16 | 2 | 0.125 | 0.125 | 8 | 1 | 0.125 | 0.125 | 4 | 0.5 | < 0.125 | 0.125 | 0.5 | 0.25 | < 0.125 | 0.125 |
| S5-84-4 | 4 | 8 | 0.125 | 0.125 | 0.5 | 0.25 | < 0.125 | 0.25 | 0.5 | 0.25 | < 0.125 | 0.125 | 0.5 | 0.25 | < 0.125 | 0.125 |
| S10-28 | 2 | 1 | 0.125 | 0.125 | 0.5 | 0.25 | < 0.125 | 0.25 | 0.5 | 0.25 | < 0.125 | 0.125 | 0.5 | 0.25 | < 0.125 | 0.125 |
| S10-42-1 | 4 | 0.25 | < 0.125 | 1 | 0.5 | 0.25 | < 0.125 | 0.25 | 0.5 | 0.25 | < 0.125 | 0.125 | 0.5 | 0.25 | < 0.125 | 0.125 |
| **S10-84-6** | **32** | **2** | **2** | **1** | **32** | **2** | **0.5** | **0.5** | **32** | **1** | **0.5** | **0.5** | **16** | **1** | **0.25** | **0.5** |

FLU, fluconazole; VOR, voriconazole; ITR, itraconazole; POS, posaconazole; PRO, propiconazole.

Table S7 Primers used in this study.

| Gene | Primer sequence (5’- 3’) |
| --- | --- |
| ITS1 | TCCGTAGGTGAACCTGCGG |
| ITS4 | TCCTCCGCTTATTGATATGC |
| *hph-F* | GATGTAGGAGGGCGTGGATATGTCCT |
| *hph-R* | GTATTGACCGATTCCTTGCGGTCCGAA |
| *qerg11*-F | GGATCTCGACGGCGGTTTTA |
| *qerg11*-R | GTGTTCGTGGTCGCTTTCAC |
| *qAfr1*-F | TGGTAATGTCGAGTACGCCG |
| *qAfr1*-R | TTTGTGGCAAGGGCAAATCG |
| *qAfr2*-F | GATCATCCAGAGGCAGACCG |
| *qAfr2*-R | TAGTTTTGCCCGCACCAGAA |
| *qMdr1*-F | TGCTGGAGCCGTTAAGACAG |
| *qMdr1*-R | CCGCAAAGAGACACTGGGAT |
| *qAfr3*-F | GCTGTCCACCTACTTTGCCT |
| *qAfr3*-R | AAGCGTTGGTGATCCTCTCG |
| *qActin1*-F | ACTGCCCAAGTAAACTCGCC |
| *qActin1*-R | TCGAGGGCGACCAACAATAG |


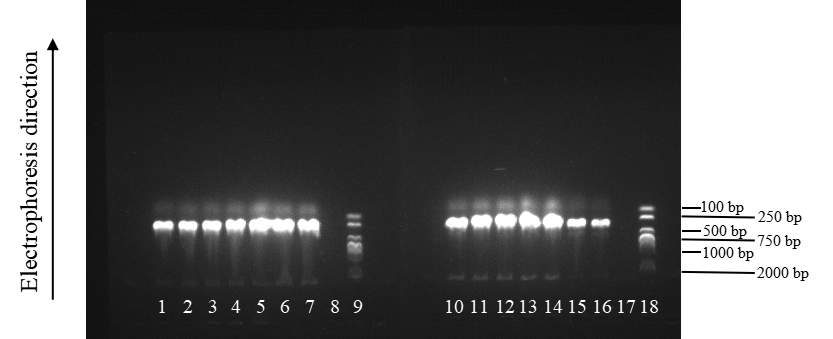


Figure S1 Agarose gel electrophoresis of the hygromycin gene amplified from C. *neoformans.* (9 and 18: DL 2000 Marker; 8 and 17, wild-type sensitive C. *neoformans*; 1-7 and 10-16: the resistant P1 C. *neoformans* isolated from soil).
